# Careless Responding Threatens Factorial Analytic Results and Construct Validity of Personality Measure

**DOI:** 10.3389/fpsyg.2019.01258

**Published:** 2019-06-14

**Authors:** Chester Chun Seng Kam

**Affiliations:** Faculty of Education, University of Macau, Macau, China

**Keywords:** careless responding, insufficient effort responding, factor analysis, construct validity, convergent validity, discriminant validity

## Abstract

The current research investigates the impact of careless responding on factorial analytic results and construct validity with real data. Results showed that inclusion of careless respondents in data analysis distorts factor loading pattern and hinders recovery of theoretical existing factors. Careless respondents also blur the distinction of theoretically distinct factors, resulting in higher inter-factor correlations. That careless responding may threaten convergent validity also receives limited support. Researchers are advised to exclude careless respondents before statistical analysis.

Research on personality relies on collection of accurate data. If a dataset includes a large number of unmotivated respondents, it becomes questionable if conclusion drawn will remain valid. Previous researchers (e.g., Hinkin, 1995) have suggested simple methods to maintain respondents' motivation, such as administering a shorter survey to reduce fatigue or designing questions that are easy to read. Another easy method is to check the existence of careless responding (also called insufficient effort responding; Huang et al., 2012) and exclude the data in analysis (Meade and Craig, 2012; Maniaci and Rogge, 2014; Huang et al., 2015; Kam and Meyer, 2015). However, checking for careless respondents has not been as common as it should be, perhaps because researchers are not fully aware of the effect of careless responding in biasing their research conclusion. It may require more empirical evidence to convince researchers of the importance to control for careless responding. Therefore, in the current research, we will investigate the potential effect of careless responding on factor loading pattern, convergent validity, and discriminant validity. We will use a popular personality measure in our illustration.

## Careless Responding

Careless responding (or insufficient effort responding) happens when participants did not pay enough attention to read survey items, did not fully process the items, did not retrieve relevant information from the memory, or did not integrate information from the memory with the items before responding (Tourangeau et al., 2000; Weijters et al., 2013). Previous research showed at least two types of careless respondents (Kam and Meyer, 2015). The first type of careless respondents randomly picked an answer in each item. This random response pattern may deflate the correlations among scale items (Kam and Meyer, 2015). The second type of careless respondents may give identical answers to each item. Variable scores may become more similar to each other, causing their inter-correlations to be inflated (Kam and Meyer, 2015). Previous research has shown that the results of careless responding on variable correlations can be unpredictable (Huang et al., 2015; Kam and Meyer, 2015). Researchers have been strongly advised to exclude careless respondents in their data before conducting statistical analysis (Meade and Craig, 2012; Maniaci and Rogge, 2014; Huang et al., 2015; Kam and Meyer, 2015; McGonagle et al., 2016). As mentioned, however, this has not been a common practice.

There have been several possible reasons why controlling for careless respondents has not been a common research practice. First, researchers may not have included a priori measure to check for careless responding. According to some research (Kam and Chan, 2018), it is best to include a priori measure rather than *post-hoc* methods to check for careless responding, because some of the *post-hoc* measures may confound with other response styles. One of these *post-hoc* measures, inconsistent response check among synonym items, may confound with participants' cognitive inability in providing consistent responses. Research study has shown that a large group of respondents have problems providing consistent answers to items with similar meaning, even when they have been careful in responding (Kam and Fan, 2018). Another *post-hoc* measure, such as agreeing with antonyms (e.g., outgoing and shy), may confound with attitudinal ambivalence (Jonas et al., 2000) or acquiescence response bias (Kam and Meyer, 2015), both of which has nothing to do with careless responding.

Second, researchers may be reluctant to exclude careless respondents because such practice will inevitably reduce sample size. Most statistical analysis has a minimum recommendation on sample size (Hinkin, 1995), and journal editors and reviewers tend to favor a study with a larger number of participants. However, such practice prevents careless respondents to be excluded, and as we will show, careless respondents have a strong biasing effect on statistical results. In this paper, we will focus on how careless respondents has the potential to distort factor loading pattern, convergent validity, and discriminant validity.

## Potential Impact of Careless Responding

We hypothesized that careless responding can possibly distort the factor loading pattern in factor analysis. When participants randomly answer survey items, item correlations may decrease, attenuating magnitude of factor loadings (Crede, 2010; Kam and Meyer, 2015). When careless participants choose identical responses throughout a survey, it will increase inter-correlation among items from different constructs, thus blurring the distinction among *different* constructs (DeSimone et al., 2018). Therefore, with the existence of careless responding, cross-loadings may become more prevalent.

We also hypothesized that careless responding may attenuate the convergent validity between self-rating and peer-rating. With the existence of careless responding in a dataset, response errors increase. Because response errors from self-rating should not correlate with peer-rating, construct correlations between self-rating and peer-rating may become weaker.

Finally, we hypothesized that careless responding may decrease discriminant validity (i.e., increase correlations) among theoretically distinct constructs. When respondents give random answers or identical answers to consecutive survey items, the distinction among various constructs decreases. When respondents give random answers (i.e., first type of careless respondents), then the scale means among various distinct constructs tend to center in the mid-point of a Likert scale (e.g., 3 in a 5-point Likert scale; Huang et al., 2015). Similarly, when respondents give identical answers to consecutive survey items (i.e., second type of careless respondents), the scale means among various distinct constructs will be similar (Kam and Meyer, 2015). The resultant effect of both types of respondents is that scores from various constructs can become more similar, even when their correlations in the population should be close to zero. This will cause theoretically orthogonal constructs to correlate more strongly with each other.

## The Current Study

The purpose of the current study is to examine the effect of careless respondent inclusion on factor loading pattern, convergent validity, and discriminant validity on a popular personality measure, HEXACO measure (Ashton and Lee, 2009). With the use of this personality scale, factor loading pattern will be examined using exploratory structure equation modeling (ESEM; Asparouhov and Muthen, 2009). ESEM has the advantage of providing model fit indices while allowing cross-loading information, thus allowing us to examine how factor loading pattern can possibly be distorted with the inclusion of careless respondents. In addition, ESEM also allow the modeling of correlations (covariances) among personality factors, and between self-rating and peer-rating, permitting us to examine the convergent validity of personality factors (between self-rating and peer-rating) and the discriminant validity between personality factors, which are supposed to have orthogonal relationship with each other.

## Methods

### Participants

Two hundred and eighteen pairs of roommates (i.e., 436 students; 289 female, 146 male, and 1 unidentified; *M*_age_ = 21.17; *SD*_age_ = 3.53) from various universities in Shanghai participated in the current study. They completed an online survey in Chinese in exchange for 100 Renminbi (around US$14) per person. The study was approved by the ethics board at the University of Macau.

### Instruments

For all items, a 5-point Likert scale (1 = *Strongly Disagree*; 5 = *Strongly Agree*) was used.

### HEXACO Personality Inventory-Revised (HEXACO PI-R)

Each participant reported their own personality using 60-item version of HEXACO PI-R (Ashton and Lee, 2009). The scale measures six dimensions of personality, namely honesty-humility, emotionality, extraversion, agreeableness, conscientiousness, and openness to experience. Each dimension included 10 items. About half of the items were reverse-keyed (six for honesty-humility and conscientiousness; four for emotionality; extraversion, and agreeableness; five for openness to experience). Cronbach's alphas were good for these self-report personality factors in the current study (0.69 for honesty-humility, 0.73 for emotionality, 0.68 for extraversion, 0.72 for agreeableness, 0.69 for conscientiousness, and 0.71 for openness). In addition, participants also reported their roommate's personality using the parallel 60-item version of HEXACO PI-R. Cronbach's alphas were good for these peer-report personality factors in the current study (0.76 for honesty-humility, 0.74 for emotionality, 0.75 for extraversion, 0.83 for agreeableness, 0.80 for conscientiousness, and 0.75 for openness). Participants completed the officially back-translated, Chinese version of the scale that has been posted in the HEXACO website (http://www.hexaco.org). The order of the self-report and the peer-report versions was counterbalanced.

### Instructed Response Items

Before starting the study, participants were informed about items that instructed them to answer a certain way (e.g., “Please answer the option Disagree for this item”), and a sample item was provided (Kam and Chan, 2018). Five of such instructed response items were included in the survey.

### Analysis Strategy

In the current study, participants who answered more than half of the instructed response items (i.e., three out of five items) correct are considered careful respondents, and all responses from careless respondents are excluded. In cases when only one member within a pair is excluded (due to careless responding), data from excluded members would be analyzed using full information maximum likelihood (FIML) analysis. Using this criteria, 204 pairs of roommates (from 377 respondents; 255 female and 122 male) were included in the careful respondent sample. All data were analyzed using Mplus 7.1 (Muthén and Muthén, 1998-2012), with the complex analysis option to account for data non-independence (i.e., individuals nested within each roommate pair). Although all items were measured on an ordinal (5-point Likert) scale, Rhemtulla et al. (2012) showed that ordinal scales can be treated as continuous when the number of categories is five or more.

**Figure 1:**
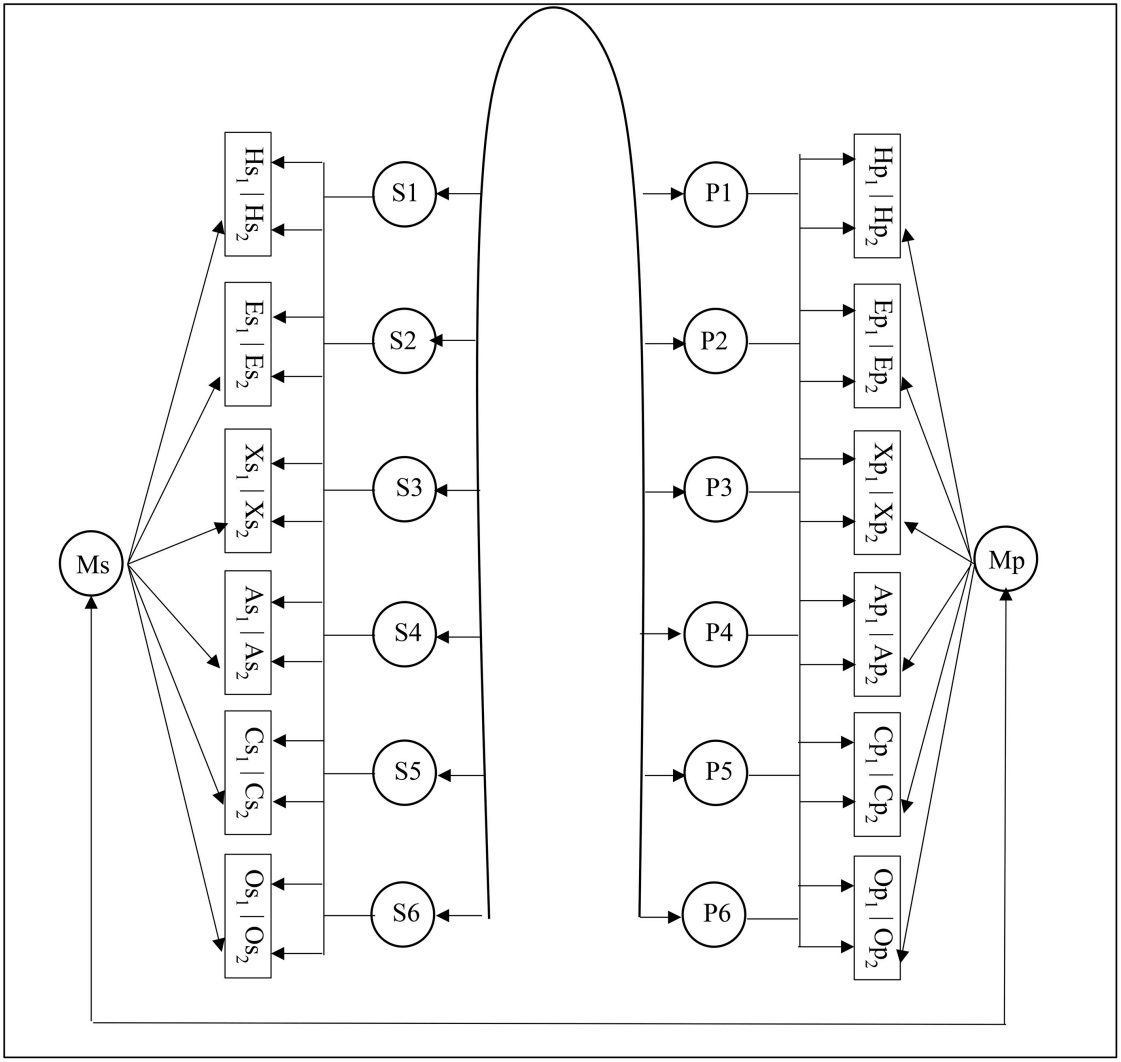
Graphic representation of the final model. All self-report personality items loaded on latent factors S1-S6. Most self-report items had major loadings on one factor and cross-loadings on other factors. Similarly, all peer-report personality items loaded on latent factors P1-P6. Most peer-report items had major loadings on one factor and cross-loading on other factors. S1-S6 and P1-P6 were allowed to correlate with each other. Reverse-keyed self-report items loaded on a method factor (Ms), and reverse-keyed peer-report items loaded on another method factor (Mp). H, honesty-humility items; E, emotionality items; X, extraversion items; A, agreeableness items; C, conscientiousness items; O, openness to experience items; s, self-report; p, peer-report; subscript 1, regular-keyed items; subscript 2, reverse-keyed items. For presentation purpose, only item groups are presented. For example, Hs_1_ represents all regular-keyed items for self-report honesty-integrity measure. The following information was not shown in this figure due to space limitation. First, self-report method factor (Ms) was allowed to correlate with peer-report personality factors (P1-P6). Second, peer-report method factor (Mp) was allowed to correlate with self-report personality factors (S1-S6).

To demonstrate the effect of careless respondent inclusion, we first analyzed the data with the entire sample. We set up an Exploratory Structural Equation Modeling (ESEM) model (with goemin rotation) that allows the self-report HEXACO factors and the peer-report HEXACO factors to be freely correlate with each other. ESEM has an important advantage over simple confirmatory factor analysis (CFA) because the former relaxes the assumption of zero cross-loadings among items, thereby improving model fit without the need of resorting to parceling strategy. Marsh et al. (2014) strongly advocated the use of ESEM to analyze personality data. In the current study, each self-report HEXACO factor was allowed to have cross-loadings with other self-report HEXACO factors, and, similarly, each peer-report HEXACO factor was allowed to have cross-loadings with other peer-report HEXACO factors. In both self- and peer-ratings, a theoretically driven six-factor HEXACO model was imposed (Figure 1). Previous research has shown the existence of method effect due to the use of reverse-keyed items in personality measures. We therefore included a method factor on reverse-keyed items for self-report HEXACO factors and another method factor for peer-report HEXACO factors, and compared this model with a model without a method factor. We expected the model with a method factor to fit better than the model without.

For the purpose of the current study, we are mainly interested in comparing the entire sample with the careful respondent only sample on two aspects: first, factor loading pattern of HEXACO factors; and second, the construct validity (i.e., convergent and discriminant validity) between self-report and peer-report of each HEXACO latent factor. For convergent validity, self-ratings and peer-rating of the same personality factors should be correlated well with each other. Therefore, we examined the strength of the correlations for the same personality factors between the entire sample and the careful respondent only sample. For discriminant validity, personality factors are theoretically orthogonal (due to the non-redundancy of the factors) and thus should not be strongly correlated. We examined the cross-correlations between personality factors and compare such correlations between the entire sample and the careful respondent only sample.

## Results

### Factor Analysis Results

The ESEM model with method factor fit significantly better than the ESEM model without method factor, in both the entire sample (Δχ^2^ = 293.67; Δ*df* = 71, *p* < 0.001) and the careful respondent sample (Δχ^2^ = 304.01; Δ*df* = 71, *p* < 0.001). Therefore, the method factor model was used. When the entire sample is analyzed, ESEM analysis failed to show clear loading pattern for all of the six HEXACO factors (Table 1). Honesty-integrity factor was missing in both self-ratings and peer-ratings, and agreeableness items did not load well in the self-rating data—only half of the items loaded on the agreeableness factor. In addition, there were a substantial number of cross-loadings in extraversion items, in both self-ratings and peer-ratings. In both types of ratings, four out of ten extraversion items cross-load with the unknown factor. Given the result of an entirely missing factor (honesty-integrity) and substantial number of cross-loadings, a researcher may conclude that the six-factor HEXACO model was only partially supported by the data.

**Table 1:** Factor loadings for entire sample.

	**Self-Rating**							**Peer-Rating**						
**Items**	**UNKN**	**E**	**X**	**A**	**C**	**O**	**Method**	**UNKN**	**E**	**X**	**A**	**C**	**O**	**Method**
h1	−0.25	0.03	0.05	0.33^***^	0.21	0.08		−0.03	−0.04	−0.01	0.34^*^	0.15	0.19	
h2	0.08	−0.13	0.05	0.30^***^	0.02	0.11		0.04	−0.20	−0.15	0.51^***^	0.06	0.18	
h3	−0.16	0.11	0.10	0.24^**^	0.24	0.10		−0.02	0.09	0.12	0.28	0.31^*^	0.17	
h4	−0.09	−0.02	0.17	0.26^**^	0.15	0.08		−0.17^*^	0.08	0.09	0.28^*^	0.03	0.15	
h5	−0.22^*^	−0.02	0.01	0.14	0.10	0.15	0.35^***^	−0.09	−0.10	0.19	0.15	0.18	0.08	0.31^*^
h6	−0.27^**^	0.03	−0.22^*^	0.02	−0.07	−0.09	0.38^***^	−0.22^**^	−0.04	−0.12	0.19^*^	0.01	−0.24^**^	0.32^***^
h7	–0.30^***^	−0.20^**^	0.04	0.03	−0.02	0.05	0.30^***^	−0.22	−0.06	0.05	0.07	0.12	0.05	0.32^***^
h8	−0.20^*^	−0.15^*^	0.05	0.11	0.06	0.11	0.19^**^	−0.01	**–0.35**	−0.19	**0.32**	0.08	0.04	0.17
h9	–0.49^***^	0.01	−0.09	0.10	−0.10	−0.02	0.31^***^	–0.47^***^	−0.04	0.03	0.30^***^	0.01	−0.07	0.27^***^
h10	−0.27^**^	0.001	0.17^*^	0.03	0.07	0.14	0.36^***^	−0.09	0.06	0.24	0.12	0.18	0.12	0.30^*^
e1	0.04	0.38^***^	−0.11	0.11	0.02	−0.05		0.11	0.46^***^	−0.05	0.03	−0.002	0.08	
e2	−0.01	0.47^***^	−0.22^*^	−0.16	0.11	0.14		0.04	0.53^***^	−0.26^**^	−0.10	0.04	0.09	
e3	0.17^*^	0.63^***^	0.13	0.03	−0.02	0.01		0.05	0.62^***^	0.06	0.11	0.06	−0.06	
e4	0.22^*^	0.43^***^	−0.01	0.21^**^	0.01	−0.07		0.29^***^	0.39^***^	−0.07	0.42^**^	−0.07	0.01	
e5	−0.14	0.51^***^	0.09	−0.003	0.10	0.05		−0.19^*^	0.67^***^	0.06	−0.03	0.08	0.03	
e6	0.03	0.57***	0.17^*^	0.06	0.05	0.07		0.004	0.53^***^	0.03	0.20	0.15	0.04	
e7	0.02	0.19^**^	–0.33^**^	−0.27^***^	0.19	−0.02	0.25^***^	−0.03	0.15^*^	–0.37^***^	−0.07	0.23^***^	−0.01	0.27^***^
e8	−0.05	0.54^***^	−0.03	−0.16	−0.07	−0.09	0.41^***^	−0.07	0.36^***^	−0.14	0.04	−0.10	−0.12	0.47^***^
e9	−0.29^***^	0.41^***^	−0.01	−0.21^**^	−0.04	−0.03	0.33^***^	−0.16	0.46^***^	−0.11	−0.09	−0.10	−0.11	0.41^***^
e10	−0.01	0.34^***^	−0.03	−0.08	−0.17	−0.10	0.45^***^	−0.05	0.32^***^	0.10	−0.10	−0.03	−0.03	0.50^***^
x1	0.10	0.11	0.65^***^	0.02	0.03	−0.04		0.05	−0.02	0.60^***^	−0.14	0.09	0.11	
x2	0.44^***^	0.003	0.26^*^	0.06	−0.16	−0.07		0.47^***^	0.06	0.17	0.17^*^	−0.12	−0.09	
x3	−0.03	0.07	0.68^***^	0.10	−0.03	0.01		−0.03	0.01	0.63^***^	−0.11	−0.02	0.12	
x4	0.65^***^	−0.07	**0.30**	−0.05	−0.13	0.01		0.72^***^	−0.04	0.37^*^	0.01	0.01	−0.01	
x5	0.47^***^	0.17^*^	0.29^*^	0.08	−0.15	−0.08		0.56^***^	0.02	0.40^*^	0.04	−0.08	−0.03	
x6	0.61^***^	−0.01	0.36^*^	−0.07	0.07	0.11		0.62^***^	−0.07	−0.23	−0.05	0.25^***^	0.02	
x7	0.29^*^	−0.07	0.28	–0.31^***^	0.03	0.19^*^	0.37^***^	0.26^*^	−0.02	0.38^***^	−0.19^*^	0.19^*^	0.10	0.30^***^
x8	−0.01	0.08	0.59^***^	−0.003	0.01	−0.04	0.38^***^	0.01	0.03	0.49^***^	0.09	0.14	0.004	0.32^***^
x9	−0.19	−0.07	0.50^***^	−0.07	−0.03	0.04	0.36^***^	−0.01	−0.02	0.56^***^	−0.07	0.01	−0.06	0.32^***^
x10	−0.01	−0.24^**^	0.42^***^	0.04	−0.003	−0.08	0.33^***^	0.01	−0.22^***^	0.46^***^	−0.03	0.04	−0.01	0.23^***^
a1	0.14	−0.18^*^	−0.08	0.66^***^	0.01	0.05		0.15	−0.09	−0.03	0.74^***^	−0.03	0.03	
a2	0.16	−0.06	−0.12	0.66^***^	−0.06	−0.03		0.09	0.04	−0.04	0.60^***^	−0.04	0.03	
a3	−0.09	0.15	0.15	0.56^***^	0.04	0.02		−0.02	0.06	0.08	0.73^***^	0.01	0.02	
a4	0.05	0.15	0.21	0.26^***^	0.06	0.02		0.05	0.11	0.20^**^	0.37^***^	0.09	−0.02	
a5	−0.14	−0.10	0.12	0.46^***^	−0.05	0.07		−0.01	0.02	−0.01	0.50^***^	0.04	0.03	
a6	−0.03	0.01	−0.15	0.59^***^	0.18^*^	−0.11		−0.08	0.04	0.09	0.63^***^	0.11	0.10	
a7	−0.24	0.08	0.11	0.21^*^	−0.28^***^	−0.06	0.31^***^	−0.28^**^	0.02	0.20	0.38^***^	−0.10	−0.15	0.38^***^
a8	−0.01	−0.04	0.16	0.14	–0.44^***^	−0.18	0.29^***^	0.01	−0.13	0.20	0.32^*^	−0.07	−0.25^**^	0.32^***^
a9	−0.28^*^	−0.01	0.09	0.28^*^	−0.06	−0.07	0.47^***^	**–0.31**	0.02	0.15	**0.33**	0.08	0.001	0.39^***^
a10	−0.20^*^	−0.12	0.05	0.29^***^	−0.02	−0.01	0.37^***^	−0.23	−0.09^*^	−0.02	0.48^***^	0.11	−0.15^*^	0.29^***^
c1	0.17^*^	−0.05	0.09	0.17^*^	0.48^***^	−0.23		0.25^**^	0.02	0.03	0.01^*^	0.64^***^	−0.07	
c2	0.33^***^	0.13	−0.10	−0.01	0.56^***^	0.02		0.33^***^	0.02	0.25	0.02	0.47^***^	0.05	
c3	0.04	0.09	0.03	0.11	0.66^***^	0.04		0.003	0.12	0.06	0.05	0.68^***^	0.20	
c4	0.30^***^	0.01	−0.14	0.17	0.49^***^	−0.05		0.33^*^	< 0.001	−0.28	−0.05	0.33^***^	0.22	
c5	−0.20^*^	−0.15^*^	0.03	−0.06	0.43^***^	−0.17	0.31^***^	−0.12	−0.02	−0.11	0.04	0.58^***^	0.03	0.30^***^
c6	−0.11	−0.26^***^	0.09	−0.01	0.18^*^	−0.01	0.49^***^	−0.03	−0.12	0.01	0.04	0.38^***^	−0.07	0.45^***^
c7	0.05	–0.41^***^	−0.03	−0.05	−0.002	−0.20^*^	0.24^***^	−0.04	−0.21^**^	−0.003	−0.13	0.42^***^	−0.20^*^	0.23^***^
c8	−0.02	0.02	0.09	0.004	0.38^***^	−0.02	0.44^***^	0.14	0.04	0.01	0.05	0.58^***^	−0.08	0.49^***^
c9	−0.01	–0.41^***^	0.05	0.02	0.15	−0.18	0.44^***^	−0.08	−0.07	0.13	0.06	0.51^***^	−0.10	0.28^***^
c10	−0.06	−0.09	0.03	−0.03	0.38^***^	−0.23^**^	0.46^***^	−0.02	−0.11	−0.11	−0.05	0.50^***^	−0.17	0.28^***^
o1	0.24^*^	0.07	−0.001	0.21^**^	0.07	0.32^***^		0.14	−0.03	−0.05	0.03	0.09	0.47^***^	
o2	0.27	0.01	−0.11	0.16	−0.03	0.48^***^		0.17	−0.03	−0.21^**^	0.04	0.07	0.66^***^	
o3	0.15	0.21^***^	0.07	0.16^*^	0.01	0.47^***^		0.01	0.09	−0.04	0.05	0.10	0.59^***^	
o4	0.26^**^	−0.004	0.32^**^	0.03	0.05	0.39^***^		0.03	0.16	0.29^**^	0.01	−0.02	0.45^***^	
o5	−0.11	0.36^***^	0.27	−0.04	0.13	0.46^***^		−0.08	0.14	0.29^*^	0.02	0.20^*^	0.40^**^	
o6	−0.10	−0.03	−0.01	−0.06	−0.10	0.50^***^	0.45^***^	−0.19	−0.05	0.06	−0.01	−0.03	0.63^***^	0.43^***^
o7	−0.10	−0.02	−0.06	−0.02	−0.03	0.24	0.36^***^	−0.18	−0.08	−0.07	0.10	0.001	0.12	0.31^***^
o8	−0.03	−0.10	−0.09	−0.13	0.03	0.28^***^	0.23^***^	−0.02	−0.13	0.03	0.03	0.19	0.22^*^	0.35^***^
o9	0.08	−0.11	0.03	−0.01	−0.11	0.45^***^	0.17^*^	0.08	−0.10	−0.01	−0.13	−0.09	0.49^**^	0.34^***^
o10	−0.003	−0.11	−0.08	0.03	−0.07	0.51^***^	0.27^***^	−0.14	−0.21^**^	0.02	0.06	−0.04	0.41^***^	0.34^***^

Loadings at 0.30 or higher are bolded. E, emotionality; X, extraversion; A, agreeableness; C, conscientiousness; O, openness to experience; method, method factor; UNKN, unknown factor.

* p < 0.05;

** p < 0.01;

*** *p < 0.001*.

In contrast, a much better loading pattern was found in careful respondent only dataset (Table 2). First, the honesty-integrity factor was discovered, particularly in the peer-rating data. Seven out of 10 items in self-rating and 9 out of 10 items in peer-rating loaded successfully on the honesty-integrity factor. Second, in each of the factors, at least seven (out of ten items) loaded successfully on its corresponding factor. Although cross-loadings still exist, it is apparently less severe in this careful respondent data than in the entire respondent data. When examining the factor loading for extraversion, most items in self-rating and all items in peer-rating load on the factor, and cross-loadings were less severe in careful respondent data. This is at stark contrast with the loading pattern for the same factor in the entire respondent data. Interestingly, the factor loading pattern appears to be better in peer-rating (i.e., less cross-loadings) than in self-rating. Overall, the result showed superior loading pattern in careful respondent data than in entire respondent data.

**Table 2:** Factor loadings for careful respondent only sample.

	**Self–Rating**							**Peer–Rating**						
**Items**	**H**	**E**	**X**	**A**	**C**	**O**	**Method**	**H**	**E**	**X**	**A**	**C**	**O**	**Method**
h1	0.60^***^	−0.01	−0.03	0.10	0.08	−0.02		0.51^**^	0.05	−0.04	0.07	0.07	0.14	
h2	0.26	−0.10	0.07	0.16	−0.02	0.17		**0.36**	−0.09	−0.09	0.21	0.01	0.27^*^	
h3	0.51^***^	0.08	0.002	0.07	0.15	−0.01		0.68^***^	0.11	−0.06	−0.02	0.19	0.05	
h4	**0.43**	−0.04	0.12	0.07	0.03	0.05		0.43^***^	0.14	−0.04	0.15	−0.06	0.06	
h5	0.41^***^	0.02	−0.02	0.03	0.01	0.04	0.37^***^	0.59^***^	−0.10	0.12	−0.02	0.02	−0.04	0.27^***^
h6	0.05	0.004	−0.28	0.03	−0.10	−0.20	0.41^***^	0.07	−0.02	−0.25^***^	0.18	−0.02	−0.18^*^	0.34^***^
h7	0.45^*^	−0.17^*^	0.04	−0.06	−0.08	−0.05	0.21^**^	0.50^***^	−0.08	−0.11	−0.05	−0.02	−0.06	0.27^***^
h8	0.36^*^	−0.14	−0.02	0.02	−0.03	0.06	0.21^***^	0.40^*^	−0.21	−0.13	0.02	0.01	0.12	0.15^*^
h9	0.21	−0.07	−0.24	0.08	−0.20	−0.25	0.35^***^	0.30^*^	−0.08	–0.32^***^	0.35^**^	−0.07	−0.09	0.28^***^
h10	0.41^**^	−0.001	0.08	−0.05	−0.03	−0.02	0.36^***^	0.62^***^	0.02	0.15	−0.08	0.01	−0.04	0.29^***^
e1	0.01	0.40^***^	−0.07	0.07	−0.01	−0.06		−0.01	0.48^***^	0.01	−0.04	0.003	0.07	
e2	−0.010	0.43^***^	−0.25**	−0.17	0.09	0.05		0.05	0.48^***^	−0.15^*^	−0.29^***^	0.03	0.05	
e3	−0.06	0.63^***^	0.14	0.07	0.05	0.02		−0.02	0.65^***^	0.04	0.11	0.05	−0.08	
e4	−0.09	0.47^***^	0.07	0.24	0.11	0.05		0.13	0.54^***^	0.13	0.11	−0.04	0.11	
e5	0.13	0.48^***^	−0.02	−0.003	0.06	−0.11		−0.01	0.55^***^	−0.13	0.09	0.07	−0.07	
e6	0.11	0.56^***^	0.11	0.08	0.04	−0.001		0.29^*^	0.55^***^	−0.03	0.02	0.09	−0.02	
e7	−0.13	0.17^*^	–0.34^**^	−0.21^*^	0.25	−0.02	0.17^*^	−0.02	0.14	–0.30^***^	−0.17	**0.30**	0.08	0.24^*^
e8	−0.04	0.58^***^	−0.02	−0.09	−0.04	−0.16	0.36^***^	−0.02	0.39^***^	−0.16	−0.02	−0.10	−0.07	0.41^***^
e9	0.16	0.43^***^	−0.09	−0.17	−0.09	−0.24	0.32^***^	−0.11	0.49^***^	−0.23^**^	−0.03	−0.08	−0.14^*^	0.44^***^
e10	−0.04	0.43^***^	0.07	−0.02	−0.07	−0.13	0.33^***^	0.11	0.41^***^	0.04	0.11	−0.06	−0.05	0.41^***^
x1	0.17	0.09	0.64^***^	−0.02	0.04	−0.05		−0.02	−0.16	0.47^***^	0.12	0.04	−0.03	
x2	−0.15	0.04	0.42^**^	0.01	−0.07	0.15		−0.06	0.17^*^	0.43^***^	0.02	−0.09	−0.002	
x3	0.20	0.04	0.62^***^	0.08	0.04	−0.06		0.16	−0.03	0.45^***^	0.31	−0.07	−0.02	
x4	−0.22	−0.05	0.49^*^	−0.09	0.25**	**0.33**		−0.14	0.03	0.73^***^	−0.13	0.03	0.07	
x5	−0.25	0.15	**0.39**	0.10	−0.04	0.13		−0.08	0.18	0.69^***^	−0.003	−0.06	−0.01	
x6	−0.11	−0.003	0.49^*^	−0.13	−0.09	0.38^*^		< 0.001	−0.02	0.57^***^	−0.18	0.25^*^	0.10	
x7	−0.04	−0.06	0.37^***^	−0.28^*^	−0.07	0.32^*^	0.31^*^	0.04	−0.05	0.46^***^	−0.19	0.18^*^	0.06	0.29^*^
x8	0.11	0.05	0.58^***^	−0.07	0.04	−0.05	0.36^***^	0.08	−0.05	0.37^***^	0.30^**^	0.09	−0.03	0.30^***^
x9	0.19	−0.08	0.44^**^	−0.01	−0.07	−0.15^*^	0.38^***^	0.03	−0.08	0.43^***^	0.21	−0.06	−0.15	0.35^***^
x10	0.09	−0.22^*^	0.44^***^	0.06	−0.07	−0.03	0.32^***^	0.14	−0.21**	0.34^***^	0.10	−0.01	−0.13	0.24^***^
a1	0.01	−0.15	−0.03	0.60^***^	0.04	0.23		0.11	0.04	0.07	0.53^**^	0.001	0.27^*^	
a2	−0.04	−0.04	−0.04	0.63^***^	−0.02	0.16		0.09	0.14	0.05	0.45^*^	−0.03	0.22^*^	
a3	0.05	0.13	0.05	0.64^***^	0.05	0.01		0.09	0.19	0.04	0.72^***^	0.02	0.18	
a4	0.05	0.11	0.18^*^	0.27^***^	0.07	0.05		−0.06	0.15	0.19**	0.49^***^	0.07	0.05	
a5	0.09	−0.15	0.04	0.45^***^	−0.13	0.02		−0.05	0.06	−0.04	0.51^***^	0.07	0.18	
a6	−0.01	0.01	−0.15	0.61^***^	0.20^*^	−0.05		−0.002	0.08	−0.02	0.75^***^	0.11	0.24	
a7	−0.01	0.11	0.02	0.34^*^	−0.25^*^	−0.16	0.35^*^	−0.01	0.03	−0.08	0.57^***^	−0.11	−0.11	0.37^*^
a8	−0.18	−0.03	0.19^*^	0.21**	–0.39^***^	−0.14	0.28^***^	−0.15	−0.04	0.16^*^	0.51^***^	−0.01	−0.12	0.30^***^
a9	0.03	−0.02	0.01	0.45^***^	−0.06	−0.17	0.37^***^	−0.01	−0.02	−0.14^*^	0.59^***^	0.06	0.08	0.38^***^
a10	0.06	−0.12	−0.01	0.36^***^	−0.02	−0.04	0.37^***^	−0.02	−0.07	−0.18^*^	0.59^***^	0.11	−0.01	0.26^***^
c1	−0.03	−0.08	0.14	0.19	0.55^***^	−0.11		0.03	−0.004	0.12	0.01	0.67^***^	−0.06	
c2	−0.03	0.09	−0.06	−0.07	0.59^***^	0.17		−0.02	0.14	0.02	−0.14	0.53^***^	0.12	
c3	0.27	0.04	−0.01	0.02	0.63^***^	0.08		0.27	0.04	0.01	0.03	0.60^***^	0.12	
c4	0.05	0.02	−0.02	0.08	0.52^***^	0.16		−0.04	0.11	0.004	−0.24	0.43^***^	0.28^***^	
c5	0.07	−0.20^*^	−0.05	−0.02	0.43^***^	−0.22	0.33^*^	0.08	−0.03	−0.16**	0.12	0.59^***^	0.02	0.28^*^
c6	0.03	–0.30^***^	−0.04	0.06	0.18^*^	−0.03	0.49^***^	−0.05	−0.12	−0.03	0.15	0.41^***^	−0.02	0.41^***^
c7	−0.04	–0.32^***^	0.11	−0.04	0.06	−0.08	0.19**	−0.02	−0.24	−0.02	0.04	0.43^***^	−0.21**	0.21^***^
c8	0.13	−0.001	0.05	−0.02	0.40^***^	−0.01	0.41^***^	0.23^**^	0.06	0.08	−0.04	0.53^***^	−0.05	0.42^***^
c9	−0.03	–0.35^***^	0.13	0.11	0.22^*^	−0.08	0.41^***^	0.13	−0.13	0.02	0.15	0.49^***^	−0.15	0.36^***^
c10	−0.02	−0.11	0.03	0.05	0.44^***^	−0.20	0.42^***^	−0.06	−0.12	−0.13	0.01	0.58^***^	−0.11	0.28^***^
o1	−0.01	0.07	−0.02	0.20^*^	0.07	0.45^***^		−0.03	−0.06	0.06	−0.04	0.13	0.50^***^	
o2	−0.04	0.06	−0.07	0.18^*^	−0.02	0.60^***^		−0.10	−0.04	−0.02	0.15	−0.02	0.76^***^	
o3	0.11	0.23**	0.02	0.15	−0.01	0.48^***^		0.01	0.05	−0.03	0.01	0.10	0.62^***^	
o4	0.07	−0.03	0.28^***^	−0.02	0.02	0.47^***^		0.10	0.12	0.25^***^	−0.04	−0.05	0.36^***^	
o5	0.31^**^	0.28**	0.07	−0.09	0.04	0.31^*^		0.23	0.03	0.17^*^	0.07	0.15	0.24**	
o6	0.21	0.05	−0.04	−0.01	−0.13	0.42^**^	0.37^***^	0.07	−0.09	−0.03	0.09	−0.03	0.60^***^	0.38^***^
o7	0.13	0.02	−0.08	0.02	−0.02	0.17	0.31^***^	0.21	−0.02	−0.13	0.07	−0.05	0.12	0.29^***^
o8	0.07	−0.05	−0.07	−0.08	0.02	0.26**	0.20**	0.13	−0.11	0.04	0.04	0.18	0.24**	0.32^***^
o9	−0.01	−0.10	−0.01	0.07	−0.11	0.50^***^	0.12	−0.15	−0.12	0.09	−0.07	−0.05	0.57^***^	0.24^***^
o10	0.16	−0.09	−0.12	0.01	−0.12	0.50^***^	0.24^***^	0.07	−0.19**	−0.001	0.13	−0.05	0.44^***^	0.29^***^

Loadings at 0.30 or higher are bolded. H, honesty–humility; E, emotionality; X, extraversion; A, agreeableness; C, conscientiousness; O, openness to experience; method, method factor.

* p < 0.05;

** p < 0.01;

*** *p < 0.001*.

Based on traditional cutoffs for fit indices (e.g., Fan and Sivo, 2007), the overall fit was somewhat inadequate in both the entire sample (χ^2^ = 11099.17, *df* = 6343, *p* < 0.001, TLI = 0.67, CFI = 0.70, RMSEA = 0.04, SRMR = 0.05) and the careful respondent only sample (χ^2^ = 10894.05, *df* = 6343, *p* < 0.001, TLI = 0.65, CFI = 0.69, RMSEA = 0.04, SRMR = 0.05). TLI and CFI were both suboptimal but RMSEA and SRMR were good. However, a large number of items can cause likelihood ratio statistics to deviate from the assumed chi-square distribution, resulting in inflated Type 1 error (Yuan et al., 2019). Shi et al. (2018) showed that both TLI and CFI are inaccurate assessments of model fit in a large model.

### Convergent Validity Evidence

In personality measures, self-ratings and peer-rating should be correlated well with each other. Therefore, we examined the correlation of each personality trait between self-rating and peer-rating in the entire sample and in the careful respondent sample. The result, shown in Table 3, showed that the correlations for the careful respondent sample tended to be stronger than those for the entire sample (except openness). Due to the non-independence between the two sets of data (i.e., the careful respondent sample comes from the entire sample), the correlations could not be statistically compared.

**Table 3:** Correlation of each personality factor between self-rating and peer-rating.

	**Entire sample**	**Careful respondent Sample**
H	−	0.46^***^
E	0.38^***^	0.44^***^
X	0.39^***^	0.48^***^
A	0.33^**^	0.41^***^
C	0.39^***^	0.53^***^
O	0.55^***^	0.52^***^

H, honesty-humility; E, emotionality; X, extraversion; A, agreeableness; C, conscientiousness; O, openness to experience.

** p < 0.01;

*** *p < 0.001*.

### Discriminant Validity Evidence

Personality factors are theoretically orthogonal (i.e., uncorrelated with each other). Therefore, we examined the discriminant validity among the personality factors in the entire sample and in the careful respondent samples. The result, shown in Table 4, showed weak but significant correlations among personality factors in the entire sample. In stark contrast, the correlations were apparently fewer significant correlations (3 vs. 7 significant correlations) among personality factors in the careful respondent sample. The only exception is the correlation between honesty-humility and agreeableness (*r* = 0.40) in peer-rating. This is not surprising given that the two personality factors are often confused with each other, and in this study, the confusion appears in peer-rating but not in self-rating (*r* = 0.23, *ns*). Therefore, the careful respondent sample showed a stronger discriminant validity evidence than the entire sample.

**Table 4:** Discriminant validity evidence among personality factors.

	**1**.	**2**.	**3**.	**4**.	**5**.
**ENTIRE SAMPLE**					
**Self-rating**					
1. UNKN	−				
2. E	0.002	−			
3. X	−0.07	−0.004	−		
4. A	0.03	0.01	0.022	−	
5. C	0.03	0.14^*^	0.09	0.05	−
6. O	0.05	0.04	0.02	0.03	0.20^**^
**Peer-rating**					
1. UNKN	−				
2. E	0.04	−			
3. X	−0.09	0.05	−		
4. A	−0.19	0.09	0.24^**^	−	
5. C	−0.14	−0.03	0.18^*^	0.25^***^	−
6. O	0.22	0.03	0.07	0.21^*^	0.19^**^
**CAREFUL RESPONDENT SAMPLE**					
**Self-rating**					
1. H	−				
2. E	−0.07	−			
3. X	−0.04	−0.25	−		
4. A	0.23	−0.12	0.17	−	
5. C	−0.02	0.10	0.01	−0.03	−
6. O	−0.09	0.09	−0.03	−0.07	0.16
**Peer-rating**					
1. H	−				
2. E	−0.02	−			
3. X	0.07	−0.001	−		
4. A	0.40^*^	−0.02	−0.02	−	
5. C	0.16	0.001	0.08	0.11	−
6. O	0.14	0.11	0.14^*^	−0.02	0.17^*^

H, honesty-humility; E, emotionality; X, extraversion; A, agreeableness; C, conscientiousness; O, openness to experience.

* p < 0.05;

** p < 0.01;

*** *p < 0.001*.

## Discussion

The primary motivation of the current article is to demonstrate that careless respondents can potentially threaten the factorial loading pattern and construct validity of personality measures, and the results of the current study confirmed this hypothesis. First, for factor analytic results, inclusion of careless respondents is likely to distort discovery of theoretically existing latent factors and cause serious cross-loading problem. Second, compared to careful respondents, inclusion of careless respondents may cause decrease in the correlations of personality factors between self-ratings and peer-ratings. Such decrease in correlations is, however, only modest in the current study. Nonetheless, the effect of such correlation attenuation may become blatant in meta-analytic research, when most studies did not exclude careless respondents in data analysis. In our knowledge, some research fields (e.g., industrial-organizational psychology) often employs meta-analysis to undiscover the “true” correlation among constructs. Third, compared to careful respondents, inclusion of careless respondents is likely to inflate correlations among theoretically distinct constructs. This result is particularly striking in the self-report data in the current study. The number of statistically significant correlations is higher in the entire sample as opposed to in the careful respondent only sample. Given these results, researchers who are interested in conducting construct validation studies should no longer ignore the effect of careless respondents in their data.

A critic may question the validity of the HEXACO scale due to the moderate relationship—rather than theoretically predicted orthogonal relationship—between two dimensions (honesty-humility and agreeableness) in peer report. However, observer reports are more likely than self-reports to have difficulty discriminating between these two dimensions (Lee and Ashton, 2006), perhaps because observers have less information to differentiate between nice people and honest people. Both HEXACO dimensions, however, showed discriminant validity with external variables (Hilbig et al., 2013), meaning that they are two distinct constructs. The moderate relationship should therefore not undermine confidence in the validity of the scale.

The current study has several limitations. First, we employed a popular personality scale among educated Chinese university students; future research should extend the generalizability of the results using other measures among less educated participants. Second, we employed ESEM analysis, and the results should generalize to exploratory factor analysis. Future research may look at the impact of careless responding in other statistical analytic techniques. Finally, we limited our investigation of convergent validity to the relationship between self-rating and peer-rating. We also limited our investigation of discriminant validity to the relationship among personality factors. Future research can further broaden the scope of our investigation.

## Data Availability

The datasets generated for this study are available on request to the corresponding author.

## Ethics Statement

This study was carried out in accordance with the recommendations of Research Services and Knowledge Transfer Office, University of Macau, with written informed consent from all subjects. All subjects gave written informed consent in accordance with the Declaration of Helsinki. The protocol was approved by the Research Services and Knowledge Transfer Office, University of Macau.

## Author Contributions

The author confirms being the sole contributor of this work and has approved it for publication.

### Conflict of Interest Statement

The author declares that the research was conducted in the absence of any commercial or financial relationships that could be construed as a potential conflict of interest.
